# Radiation induced esophageal adenocarcinoma in a woman previously treated for breast cancer and renal cell carcinoma

**DOI:** 10.1186/1756-0500-5-426

**Published:** 2012-08-09

**Authors:** Soundouss Raissouni, Ferdaous Raissouni, Ghizlane Rais, Meryem Aitelhaj, Siham Lkhoyaali, Rachida Latib, Amina Mohtaram, Fadoua Rais, Hind Mrabti, Nawal Kabbaj, Naima Amrani, Hassan Errihani

**Affiliations:** 1 Medical Oncology Department, National Institute of Oncology, Rabat, Morocco; 2 EFD- hepato-gastro-enterology, Ibn Sina University Hospital, Rabat, Morocco; 3 Radiology department, National Institute of Oncology, Rabat, Morocco; 4 xRadiotherapy department National Institute of Oncology, Rabat, Morocco

**Keywords:** Esophageal cancer, Radiation induced, Breast cancer, Renal cell carcinoma, Multiple neoplasm

## Abstract

**Background:**

Secondary radiation-induced cancers are rare but well-documented as long-term side effects of radiation in large populations of breast cancer survivors. Multiple neoplasms are rare. We report a case of esophageal adenocarcinoma in a patient treated previously for breast cancer and clear cell carcinoma of the kidney.

**Case presentation:**

A 56 year-old non smoking woman, with no alcohol intake and no familial history of cancer; followed in the National Institute of Oncology of Rabat Morocco since 1999 for breast carcinoma, presented on consultation on January 2011 with dysphagia. Breast cancer was treated with modified radical mastectomy, 6 courses of chemotherapy based on CMF regimen and radiotherapy to breast, inner mammary chain and to pelvis as castration. Less than a year later, a renal right mass was discovered incidentally. Enlarged nephrectomy realized and showed renal cell carcinoma. A local and metastatic breast cancer recurrence occurred in 2007. Patient had 2 lines of chemotherapy and 2 lines of hormonotherapy with Letrozole and Tamoxifen assuring a stable disease. On January 2011, the patient presented dysphagia. Oesogastric endoscopy showed middle esophagus stenosing mass. Biopsy revealed adenocarcinoma. No evidence of metastasis was noticed on computed tomography and breast disease was controlled. Palliative brachytherapy to esophagus was delivered. Patient presented dysphagia due to progressive disease 4 months later. Jejunostomy was proposed but the patient refused any treatment. She died on July 2011.

**Conclusion:**

We present here a multiple neoplasm in a patient with no known family history of cancers. Esophageal carcinoma is most likely induced by radiation. However the presence of a third malignancy suggests the presence of genetic disorders.

## Background

Esophageal cancer is the sixth most common malignancy worldwide with an estimated incidence of over than 300,000 new cases in 2011 [1]. It is more frequent in males with a current male-to-female ratio estimated 7-10: 1 [2]. Several meta-analyses have shown that tobacco and alcohol increase the risk of esophageal cancer especially the squamous cell histology [3,4]. The role of radiation as a risk factor is not well established. Evidence from previous case reports and cohort studies raised the possibility of a relation between radiation therapy and esophageal cancer [5,6]. There is a particular interest in the late side effects of radiation therapy for breast cancer because of the large number of women who receive such treatment each year [7]. In a large multicenter retrospective series examining the risk of secondary non hematological malignancies in 376,825 breast cancer survivors, there were 3,301 patients with potentially radiotherapy-associated cancers, among them 343 patients presented esophageal carcinoma with a standardized incidence ratio (SIR) of 1.44 [8]. Another large cohort of 220,806 women of the Surveillance, Epidemiology, and End Results (SEER) Program focusing on esophageal cancer after local breast cancer, diagnosed and treated between 1973 and 1993, showed that the SIR of esophageal cancer after radiation therapy was 54 percent higher than in the general population [5].

Second malignancies reported to be associated with renal cell carcinoma (antecedent, concurrent or subsequent) include bladder, prostate, rectal, and lung cancer as well as non-Hodgkin’s lymphoma and melanoma [9]. Breast cancer also reported to be associated with renal cancer. In a series of 8,667 patients treated for renal cell carcinoma collected in the Swedish family database; 677 had a second primary malignancy with SIR of 1.55; there were 44 breast cancers with SIR 1.69. However, breast cancer was not associated with an increased risk of developing upper gastro-intestinal track carcinomas [10].

Second primary malignancies among cancer survivors account for 16% of all cancers. Few data currently exist regarding the molecular mechanisms for second primary cancers and other late outcomes after cancer treatment [11,12].

This is a report of subsequent three malignancies, breast cancer, renal cell carcinoma and potentially radiation induced esophageal adenocarcinoma.

### Case report

**Figure 1:**
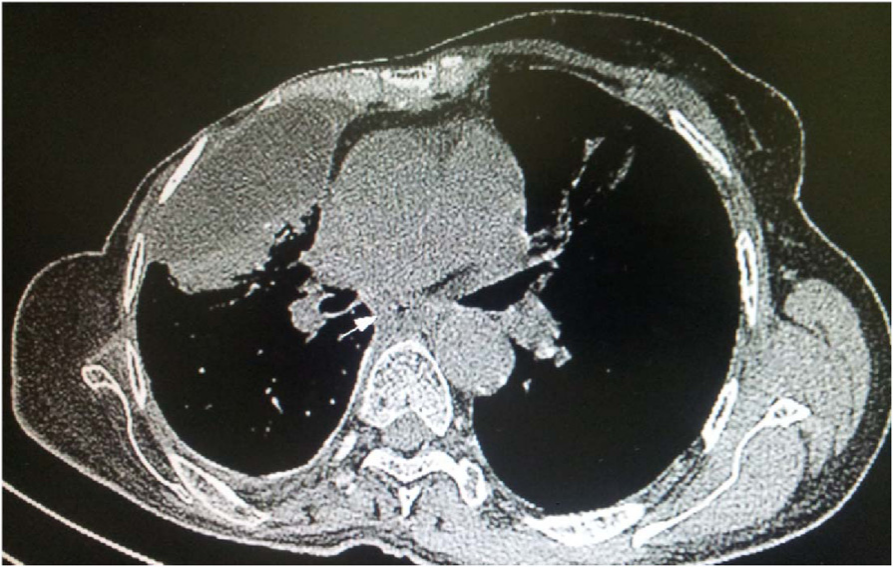
CT scan of circumferential thickening on thoracic esophagus.

**Figure 2:**
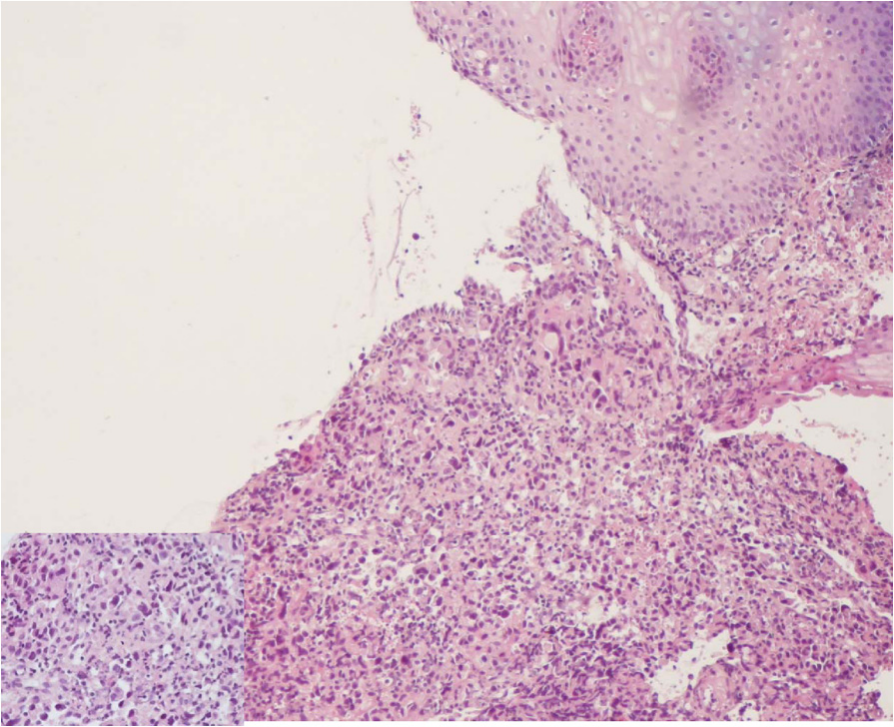
Adenocarcinoma with dissociated cells (HE, Gx100 and Gx400).

**Figure 3:**
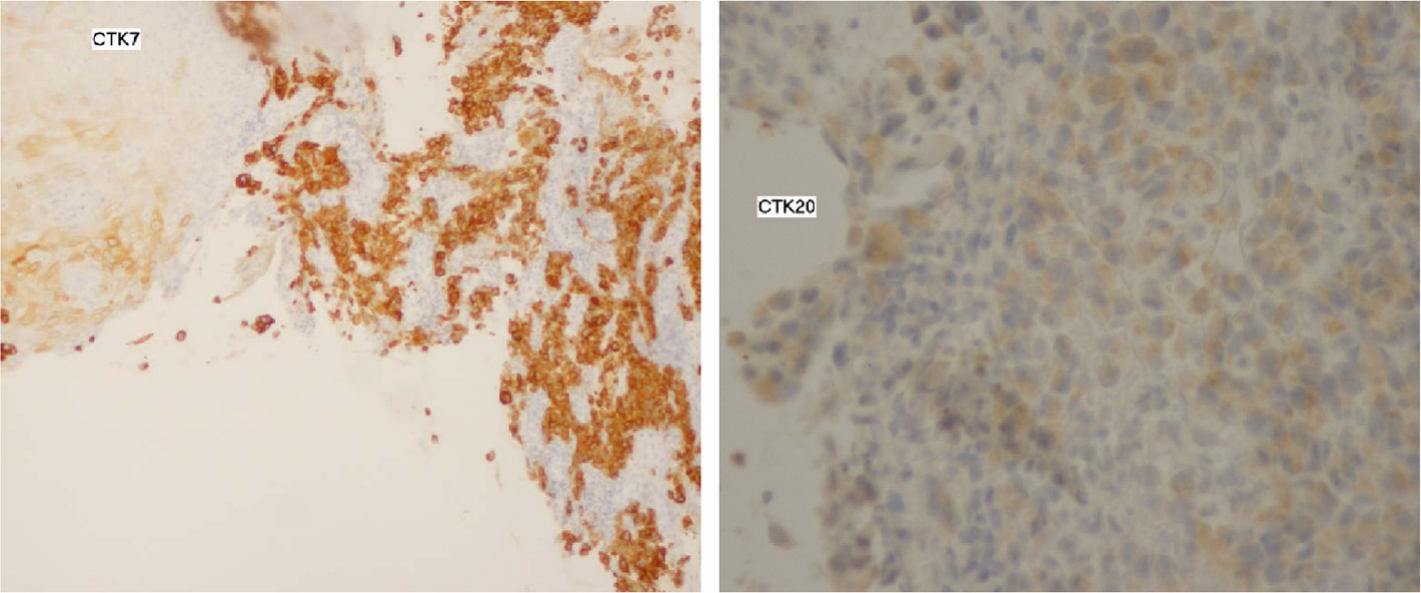
Immunolabelling CK7 and CK20 positive (Gx400).

A 56 year-old non-smoking north African woman, with no familial history of cancer, followed at the National Institute of Oncology of Rabat Morocco since March 1999 for breast cancer in premenopausal setting; presented in consultation on January 2011 with dysphagia. Her past medical history was not significant for any gastrointestinal disorders or alcohol intake. Her initial breast cancer was ductal infiltrating carcinoma of the two lower quadrants of the right breast, classified T4b N2 M0 according to TNM classification adopted by the AJCC, grade 2 of SBR (Scaff Bloom and Richardson), with vascular invasion and positive hormone receptors. She underwent modified radical mastectomy then 6 courses of adjuvant chemotherapy with CMF regimen cyclophosphamide 500 mg/m2 metotrexate 500 mg/m2 and 5FU 500 mg/m2. Adjuvant radiotherapy then was delivered 50 grays to the right breast, upper clavicular and the inner right mammary nodes. The patient received also 12 grays radiation to pelvis as castration. No adjuvant oral hormone therapy was given. Treatment achieved on August 1999. On April 2000 an incidental right renal mass, measuring 6 cm, discovered by abdominal ultrasound. Enlarged right nephrectomy was performed and the diagnosis of clear cell carcinoma of the kidney was done. On 2007, the patient presented local breast recurrence and distant relapse to bone and the pleura confirmed by a biopsy of skin nodules in mastectomy area. Both ER and PR were positive (90% and 100% respectively). HER 2 was negative. Chemotherapy with AC regimen as first line treatment (doxorubicin 60 mg/m2 and cyclophosphamide 600 mg/m2 was given with progression after 3 cycles. Docetaxel/capecitabine regimen was given as second line treatment for a total of 8 courses. A partial response then stabilization were achieved. Letrozole was given as maintenance therapy with ibandronate. Disease was stable until October 2010 where the patient presented bone progression. Patient was given Tamoxifen as second line hormonotherapy and remains stable. On January 2011 she presented dysphagia. Oesogastric endoscopy showed middle un-crossable esophagus stenosing mass. CT (computed tomography) scan showed the esophageal stenosis and stable pleural and lung breast cancer metastasis (Figure 1). Biopsy of the esophageal mass revealed an adenocarcinoma with dissociated cells (Figure 2). Immunochemistry was negative for hormone receptors and HER 2. Cytokeratines 7 and 20 were positives (Figure 3). No evidence of distant metastases or breast cancer progression on CT scan and markers was detected. Palliative brachytherapy to esophagus was delivered. Patient presented dysphagia due to progressive disease 4 months later. Jejunostomy was proposed but the patient refused any treatment. She died on July 2011 due to a esophageal progressive disease.

## Discussion

Experimental data has proven the role of radiation in developing esophageal carcinoma, induced by continuous gamma irradiation [13]. Also based on data from the cohort of Japanese survivors of the atomic bombings of Hiroshima and Nagasaki, esophageal cancers have been conclusively related to ionizing radiation exposure [14]. In human, the first case described was reported by Slaughter in 1957, 27 years of latency after radiation. It was of adenocarcinoma histological subtype [15]. Since then case reports described mainly the squamous cell carcinoma subtype [16]. In two previous large cohorts reporting data from the SEER, published in 1999 and 2006, there was an increased risk of developing esophageal cancer after breast cancer radiation [5,17]. In the first report, the risk increased with time, reaching a standardized incidence ratio of 5.42 for esophageal squamous cell carcinoma 10 years after radiotherapy. A less definitive increasing trend was evident for esophageal adenocarcinoma; the relative risk after 10 years was 4.22 [5]. In the second report, women treated with radical mastectomy and radiation had an estimated relative risks of 2.83 (95% confidence interval: 1.35, 5.92) and 2.17 (95% confidence interval: 1.67, 4.02) for squamous cell esophageal cancer at 5–9 and ≥10 years, respectively. No significant increase in risk was found for adenocarcinomas. In the same report esophageal carcinoma risk increases only for the upper (cervical) and middle (thoracic) third of the esophagus. In contrast, the risk for the lowest (abdominal) third of the esophagus was only not significantly increased [17]. Similar data were obtained from Dutch and Scandinavian registry cohorts [8,18].

Our patient presented adenocarcinoma of the middle esophagus. She fits some criteria of radiation induced cancers as previously described by Chudecki in early seventies, which are: history of previous irradiation, cancer occurring within the irradiated area and a long latent interval between irradiation and development of cancer that is over than 10 years in our case [19]. However the presence of a third malignancy (renal cell carcinoma) concurrent to breast cancer could suggest the presence of genetic disorders predisposing to such late side effects of radiation.

There are several genetic syndrome associated with multiple neoplasms, such as BRCA mutation, Li-Fraumeni syndrome and Cowden disease [11]. Syndrome-associated cancers usually develop at younger-than-usual ages, associated with a family history of developing metachronous tumors at high frequencies throughout life [20,21]. However neither the clinical presentation of our patient, nor her familial histories are typical of one of those known syndromes.

Not all previously radiated patients or given chemotherapy experience second malignancies. Late effects of treatments may be modified by genetic penetrance, interaction between gene and environment and genes interactions [22].

Individual variations of pharmacodynamics and pharmacokinetics of drug metabolizing enzymes such as glutathione S-transferase, cytochrome P450s and thiopurine methyltrans-ferases, may influence the occurrence of therapy-related malignancies [23]. In a series published by Relling and al, of children affected with leukemia and treated with prophylactic cerebral radiotherapy, children who had genetic defect of catabolism of thiopurine and high metabolism of thioguanine were more likely to present secondary brain tumors [24]. Also there are few data supporting the role of polymorphism of DNA repair in cancer related treatments susceptibility [25]. Other factors that may influence the occurrence of radiation related cancers are genomic instability, epigenetic phenomena and bystander effects [26]. In our context we couldn’t realize genetic and pharmacogenic researches because they are not available. Some charred etiologic factors such as tobacco and alcohol intake are known to be associated with multiple cancers as lung, bladder, upper gastro-intestinal tract, breast, pancreas and kidney [11]. Our patient hadn’t been exposed to such factors. Concerning the association of esophageal cancer with renal carcinoma, it was rarely reported. In a large cohort of 766 renal cell carcinomas, where 118 patients had second malignancies, only 3 had cancer of the upper gastro-intestinal tract [27]. In other large series of the Mayo clinic in 2,722 patients with renal cell carcinoma, the papillary carcinoma histology was associated significantly with multiple neoplasms [9].

## Conclusion

We report here a rare case of secondary esophageal carcinoma in a previously treated patient for breast cancer and renal cell carcinoma. The isolation of one causative factor leading to cancer is difficult. We suggest that esophageal carcinoma is more likely radiation induced. However the occurrence of three subsequent cancers suggest strongly the presence of genetic susceptibility. Careful patient selection, thorough treatment planning and modern radiation equipment could obviously reduce the dose to the surrounding tissues and thus decrease the incidence of secondary malignancies radiation induced in predisposed populations.

### Consent

Written informed consent was obtained from the patient’s next of kin for publication of this case report and any accompanying images. A copy of the written consent is available for review by the Editor-in-Chief of this journal.

## Competing interests

The authors report no conflicts of interests. The authors alone are responsible for the content and writing of the paper. Authors have equally contributed to this paper.

## Authors’ contribution

SR was involved in the management of that patient, the analysis of the data and the literature research and wrote the manuscript. FR, NK and NA were involved in the endoscopic diagnosis and management of nutritional support. GR was involved in the management of the patient and revision of the manuscript. MA, SL and AM helped with the literature research. RL performed radiological diagnosis. F R helped with the final editing of the manuscript. HM and HE approved the treatment and analyzed the literature data. All authors read and approved the manuscript.
